# Survey self-report of rheumatoid arthritis and treatments versus specialist clinician confirmation

**DOI:** 10.1186/s41927-024-00425-3

**Published:** 2024-10-09

**Authors:** Shamil Jugnundan, Gabriela Schmajuk, Laura Trupin, Paul D. Blanc

**Affiliations:** 1 Division of Occupational Environmental and Climate Medicine, University of California San Francisco, San Francisco, CA 94143-0843 USA; 2 Department of Medicine, San Francisco Veterans Affairs Health Care System, San Francisco, CA USA; 3 Division of Rheumatology, University of California San Francisco, San Francisco, CA USA

## Abstract

**Objective:**

To assess agreement between patient survey report and physician recorded arthritic conditions and medication use in order to validate population-based epidemiologic approaches to auto-immune arthritic conditions.

**Methods:**

Rheumatologists in the U.S. Appalachian region recruited men 50 years or older with a confirmed rheumatoid arthritis (RA) diagnosis. For each participating patient, the treating specialist completed a brief chart abstraction that included rheumatic diagnosis and corresponding treatment. Patients participated in a telephone interview using the same questionnaire as applied in a larger random digit dial survey that queried arthritis diagnosis and treatment. We assessed patient-clinician agreement with the Prevalence Adjusted and Biased Adjusted Kappa (PABAK) statistic.

**Results:**

We included 36 patient-clinician dyads in this analysis. All clinicians and patients concurred in the RA diagnosis (PABAK = 1). For concomitant systemic lupus and scleroderma, we observed generally concordant responses (PABAK 0.89 and 1, respectively). For medication use, for hydroxychloroquine or sulfasalazine was associated with the lowest PABAK (0.39), intermediate values for methotrexate and for the “other conventional synthetic DMARDs” category (0.67), and with the highest agreement PABAK value for the “biologic DMARD or JAK 2 inhibitor” category (0.89).

**Conclusion:**

Survey-based self-report of RA offers a useful approach in epidemiological investigation. This is particularly relevant to population-based approaches to autoimmune arthritis related to occupational and environmental factors.

**Supplementary Information:**

The online version contains supplementary material available at 10.1186/s41927-024-00425-3.

## Significance and innovations

Patient survey reported diagnosis of RA concurs with specialist diagnosis.

Patient survey reported diagnoses of concomitant arthritis diagnoses and medication treatments are less strongly concordant with clinician reported information.

Survey based epidemiologic investigation of RA offers an opportunity to study environmental and occupational disease etiology.

**Supplementary Information:**

The online version contains supplementary material available at 10.1186/s41927-024-00425-3.

Rheumatoid arthritis (RA) is an autoimmune disorder that affects 1.3 million people in the United States [1]. A systemic autoimmune disease, RA is characterized by inflammatory arthritis and extra-articular involvement. Consistent with its clinical complexity, persons with RA may also carry diagnoses of other autoimmune conditions or other forms of arthritis. Moreover, the myriad pharmacologic treatment modalities and stepwise approach to treatment in RA can lead to discordance between clinicians’ and patients’ understandings of current and past therapeutic regimens. Specifically, the extent to which patient-reported responses when surveyed about RA and related systemic autoimmune diagnoses and medication use correspond to physician reported information is not well-established.

These issues are particularly germane to survey-based epidemiologic investigations of RA in association with external risk factors. We have used such survey techniques effectively in studying coal mining and other work-related sources of silica exposure in RA [2–4]. As an adjunct to our study of the coal mining regions of Appalachia, we used data from a survey of RA patients recruited from physicians’ practices and responses from those physicians to assess the agreement between patient self-reported arthritis conditions and treatments and corresponding treating clinician reports.

## Materials and methods

We collected the data analyzed as part of a larger study of arthritis in selected counties in the Appalachian region of the inland, mid-Atlantic USA. In the larger study, men aged 50 years or older were recruited via random digit dial to participate in a telephone survey about employment and health, with particular attention to diagnosis and treatment of RA [3]. The recruitment was limited to males given its occupational focus on miners and other silica-exposed workers. The present study used information derived from this survey instrument to obtain information from male patients aged 50 years or older with physician-confirmed RA diagnoses who were recruited via rheumatology practices in the same region as the larger study. For each participating patient, the treating rheumatologist completed a brief chart abstraction instrument that included rheumatic and other autoimmune diagnoses and RA medication treatment, including use of disease modifying anti-rheumatic drugs (DMARDs). The chart abstraction form, developed for this study, is provided in Supplemental Material File A. Similarly, we surveyed participating patients about the etiology of their arthritis; whether they believed their arthritis was secondary to RA, systemic lupus erythematosus (SLE), or scleroderma (multiple etiologies could be reported, including non-autoimmune arthritis). We also elicited their reports of their treatment regimen. In the patient survey, individual drugs were queried by name, including both the generic and brand names for each medication. For the physician instrument, we elicited treatments including: methotrexate; hydroxychloroquine or sulfasalazine; biologic DMARD or JAK2 inhibitors as a group (listing the examples etanercept, alalimumab, infliximab, golimumab, certolizumab, tocilizumab, abatacept, rituximab, or tofacitinib); and a category of other conventional synthetic DMARDs (listing the examples azathioprine, leflunomide, minocycline, tacrolimus, or mycophenolate). The last three medications listed in the “other conventional synthetic DMARD” group were not included in the patient survey because their use in the treatment of inflammatory arthritis is rare. The survey battery was developed for this study; the items are included in Supplemental Material File B.

We recruited patients from five rheumatology practices in Pennsylvania, West Virginia, Kentucky, and Tennessee. A total of 50 patients expressed interest in the study and were eligible (based on age, county of residence, and RA diagnosis); 36 consented to chart review and completed a telephone interview. Data collection occurred between January 2020 and June 2021. The study was approved by the University of California, San Francisco Institutional Review Board (IRB) in accordance with the Declaration of Helsinki and all patients provided written consent to participate in the study. Four of the five practices relied on the UCSF IRB and one established its own protocol under the auspices of its institutional IRB, following the same procedures as the main protocol.

We included 36 patient-clinician dyads in this analysis. In total, 11 physicians from seven separate medical practices contributed to this recruitment, with participating patients ranging from one to seven per physician. We compared each patient participant responses to the corresponding responses from their clinician. We calculated percent agreement for each question by calculating the number of clinician vs. patient responses to each question that were concordant (i.e. both replied ‘yes’, or both replied ‘no’) as a proportion of the total number of patient-physician pairs. We used the Prevalence Adjusted and Biased Adjusted Kappa (PABAK) statistic to calculate the inter-observer agreement between patient and clinician given the low proportions of certain diagnoses and treatments [5]. Although the standard Kappa statistic commonly is employed in assessing the agreement, its value can be highly dependent on prevalence; the PABAK was developed to address this limitation [6]. 

## Results

All patient participants and physicians responded affirmatively that their arthritic condition was due to RA (PABAK = 1) (Table 1). When queried as to whether the arthritis was also due to SLE or scleroderma, we observed generally concordant responses (PABK 0.89 and 1, respectively). For medication use, hydroxychloroquine or sulfasalazine was associated with the lowest PABAK (0.39), intermediate values for methotrexate and for the “other conventional synthetic DMARDs” category (0.67), and the highest PABAK value for the “biologic DMARD or JAK 2 inhibitor” category (0.89) (Table 1).

**Table 1:** Clinician vs. patient responses regarding rheumatic conditions and treatment

	Clinician Yes *N* (%)	Patient Yes *N* (%)	Both Yes *N*	Clinician only, Yes *N*	Patient only, Yes *N*	Both No *N*	Agree (%)	PABAK
**Arthritis Diagnosis**								
RA	36 (100%)	36 (100%)	36	0	0	0	100	1
SLE	0	2 (6%)	0	0	2	34	94	0.89
Scleroderma	1 (3%)	1 (3%)	1	0	0	35	100	1
**Medication Prescribed**								
Methotrexate	29 (81%)	29 (81%)	26	3	3	4	83	0.67
HCQ or Sulfasalazine	22 (61%)	21 (58%)	16	6	5	9	69	0.39
Other conventional synthetic DMARD	9 (25%)	9 (25%)	6	3	3	24	83	0.67
Biologic DMARD or JAK 2 inhibitor	22 (61%)	22 (61%)	21	1	1	13	94	0.89

PABAK = Prevalence Adjusted and Biased Adjusted Kappa; RA = Rheumatoid arthritis; SLE = systemic lupus erythematosus; HCQ = Hydroxychloroquine; DMARD = Disease Modifying Anti-Rheumatic Drug; Other Synthetic DMARD = Azathioprine or Leflunomide (patents and physicians surveyed); Minocycline, Tacrolimus, or Mycophenolate (only physicians surveyed); Biological DMARD = Etanercept, Alalimumab, Infliximab, Golimumab, Cerulizumab, Tocilizumab, Abatacept, or Rituximab. JAK2 Inhibitor = Tofacitinib

## Discussion

Our findings of concordance between self-reported RA and a corresponding specialist’s diagnosis supports the validity of patient-reported ascertainment for this condition, an epidemiologic approach that can be critical to the investigation of occupational and environmental factors in autoimmune inflammatory disease. Self-report of other co-morbid arthritis conditions may be less reliable.

Concordance varied among medication classes, with hydroxychloroquine or sulfasalazine manifesting the lowest PABAK value. Because we queried patient participants for medications by both generic and by trade names, the failure to identify a treatment is not easily attributable to confusion over name recognition. For the conventional synthetic DMARDs, we omitted 3 treatments that were presented to the specialists, such that this could account for some of the discordance, although use of these medications would be very unlikely. The observed discordance also may be due to a lack of patient education about and understanding the purpose of particular medications, although the agreement for the biologic DMARD/JAK-2 group was substantial. Another source of discordance may be that, although a clinician prescribed a certain medication, the patient may not actually have been dispensed the medication from the pharmacy or, even if dispensed, embarked on a course of treatment [7]. In that regard, the patient participants’ reports of their current treatment regimen may be more accurate than reflected in the medical records.

We acknowledge that an important limitation of this analysis is that it only includes persons with a rheumatologist-confirmed diagnosis of RA. Future work addressing clinician vs. self-reported diagnosis should include individuals with osteoarthritis (OA) or other forms of arthritis. Persons without an established diagnosis of RA may yield less accurate information, for example, believing that they have RA when in fact they have a different type of arthritis, such as misreporting OA as RA. Although the prescribing component of this study would not be relevant to that question, our findings in terms of diagnostic agreement cannot be generalized to a population with other forms of arthritis. Although the PABAK addressed agreement given small numbers of observations, it does not obviate limitations to generalizability, saliently, agreement within a female study population where concomitant SLE might be more common. Overall, the relatively small study size should be kept in view, tempering interpretation of our findings. Another limitation to generalizability is that, because our study focused on risk among miners and other laborers with silica exposure, our analysis was limited to men, even though RA is predominantly a condition diagnosed among females.

Because the clinical extraction form did not have specific options for comorbid psoriasis or gout, we could not systematically analyze clinician vs. patient reported diagnoses for those conditions. This may be of particular interest for gout. One study observed that gout self-report was significantly more frequent with RA comorbidity than predicted by chance observation [8]. Moreover, a linguistic analysis has suggested that lay terminology as applied to gout may be problem-ridden [9]. Our clinical data extraction form also did not include joint specific involvement, precluding analysis of this topic. Our survey instrument did not replicate the National Health Interview Survey arthritis item nor the far more detailed Connective Tissue Disease Screening Questionnaire. In that context, it is interesting to note the findings of a study that utilized data from the United States Health and Retirement Study, comparing self-reported RA against Medicare claims. That study found limited agreement between self-report and confirmed diagnosis by various algorithms (the kappa statistic ranged from 0.07 to 0.15) [10]. Finally, our survey instrument did not assess health literacy. It is possible that health literacy may have contributed selection effects in study participation.

In summary, survey-based self-report of RA and RA treatments offers a useful approach in epidemiologic investigation. This can be applied effectively to address population-based approaches to autoimmune arthritis related to occupational and environmental factors.

## Electronic supplementary material

Below is the link to the electronic supplementary material.

Supplementary Material 1

Supplementary Material 2

## Data Availability

The datasets during the current study are not publicly available due to the human research approval received but are available (anonymyzed) from the corresponding author on reasonable request.

## Declarations

### Clinical trial number in the manuscript

Clinical trial number: not applicable.
