# Supplementary material for: Survey self-report of rheumatoid arthritis and treatments versus specialist clinician confirmation
Source: BMC Rheumatol. 2024 Oct 9;8:51. doi: 10.1186/s41927-024-00425-3 (PMC11462809; doi:10.1186/s41927-024-00425-3)
Supplement: Supplementary file 1 — Supplementary Material 1 [file 41927_2024_425_MOESM1_ESM.docx]

Work and Arthritis Study Chart Abstraction Form

Physician name: Date completed:

Month/Day/Year

Name and title of person completing this form:

# Patient Information

Name: DOB:

First Last Month/Day/Year

Diagnosed with RA in year: (ok to approximate) 🞏 check if unknown

(e.g., 2003)

# Does this patient have any of the other following diagnoses?

| SLE (lupus) 🞏 yes | 🞏 no | 🞏 unknown |
| --- | --- | --- |
| Systemic sclerosis 🞏 yes | 🞏 no | 🞏 unknown |
| Other autoimmune condition 🞏 yes | 🞏 no | 🞏 unknown |

If yes, which one(s)?

# Serologic status

RF positive (or level > 20 IU/mL) ………….….🞏 yes 🞏 no 🞏 unknown Anti-CCP positive (or level > 20 units) ...……..🞏 yes 🞏 no 🞏 unknown

**Joint erosions by x-ray** (attributed to RA, in any joint, ever): 🞏 yes 🞏 no 🞏 unknown

# Has this patient ever been prescribed any of the following medications for RA or another condition checked above (prescribed by any provider)?

🞏 yes 🞏 no 🞏 unknown Prednisone or other systemic glucocorticoid

🞏 yes 🞏 no 🞏 unknown Methotrexate

🞏 yes 🞏 no 🞏 unknown Hydroxychloroquine or Sulfasalazine

🞏 yes 🞏 no 🞏 unknown Other conventional synthetic DMARD (e.g., leflunomide, azathioprine,

minocycline, tacrolimus, mycophenolate)

🞏 yes 🞏 no 🞏 unknown Biologic therapy or JAK inhibitor (i.e., etanercept, adalimumab, infliximab, golimumab, certulizumab, tocilizumab, abatacept, rituximab, tofacitinib)
