# Supplementary material for: Survey self-report of rheumatoid arthritis and treatments versus specialist clinician confirmation
Source: BMC Rheumatol. 2024 Oct 9;8:51. doi: 10.1186/s41927-024-00425-3 (PMC11462809; doi:10.1186/s41927-024-00425-3)
Supplement: Supplementary file 2 — Supplementary Material 2 [file 41927_2024_425_MOESM2_ESM.docx]

**Diagnosis and Medication Survey Items**

|  | In general, would you say your health is excellent, very good, good, fair, or poor? | excellent 1 very good 2 good 3 fair 4 poor 5 no answer/don’t know dk |
| --- | --- | --- |
|  |  |  |
|  | Are you limited in any way in any activities because of a long-term physical condition? **(do not count retirement as a long-term health problem)** | yes 1 no 2 don’t know/refused dk |
|  |  |  |

|  | | | Has a doctor, nurse, or other health professional EVER told you that you have arthritis? | yes 1 no 2 don’t know/refused dk | |
| --- | --- | --- | --- | --- | --- |
|  |  | | |  |  |
|  | **(if = yes, ask:)** | | |  |  |
|  | **yes no dk/ref** | | | |  |
|  | a. Was this osteoarthritis (os-tee-oh-arth-right-us) or degenerative arthritis? 1 2 dk | | | |  |
|  | b. Was this rheumatoid (rue-ma-toyed) arthritis? 1 2 dk | | | | |
|  | c. Was this arthritis due to psoriasis (sor-eye-ah-sis)? 1 2 dk | | | | |
|  | d. Was this arthritis due to gout? 1 2 dk | | | | |
|  | e. At what age did your arthritis first start? age: ___________   no answer/refused dk | | | |  |

|  | | Has a doctor, nurse or other health professional EVER told you that you have any of the following medical conditions? **(read in order)** |  |
| --- | --- | --- | --- |
|  | **yes no dk/ref** | | |
|  | a. Lupus (LOOP-us) or SLE? 1 2 dk | | |
|  | b. Scleroderma (sklare-oh-DERM-ah)? 1 2 dk | | |

|  | | Does pain, swelling, stiffness or aching regularly affect your **(read in random order)**? |  |
| --- | --- | --- | --- |
|  | **yes no dk/ref** | | |
|  | a. Hands or wrists? 1 2 dk | | |
|  | b. Hips? 1 2 dk | | |
|  | c. Knees? 1 2 dk | | |
|  | d. Back? 1 2 dk | | |
|  | e. Neck? 1 2 dk | | |
|  | f. Ankles or feet? 1 2 dk | | |

|  | **(if yes to any a-f, ask:)** |  |
| --- | --- | --- |
|  | For the pain, swelling or stiffness you just identified, have you ever been given . . . **(read in random order)**? | |
|  | **yes no dk/ref** | |
|  | a. Prednisone (PRED-nuh-zone) or steroid pills? 1 2 dk | |
|  | b. Steroid injections into your muscles or joints? 1 2 dk | |

| **(if diagnosis and symptoms or treatment with prednisone) ask:** | |  |  |
| --- | --- | --- | --- |
| 15. In the past 10 years have you ever been given any of the following medicines . . . **(read in random order)**? | |  |  |
| **yes no dk/ref** | |  |  |
| a. Methotrexate (meth-oh-TREX-ate), Rheumatrex (ROOM-ah-trex), Trexall (TREX-all), Otrexup (oh-TREX-up), or Rasuvo (rah-SOOV-oh)1 2 dk | |  |  |
| b. Sulfasalazine (sulf-ah-SAL-uh-zeen) or Azulfidine (ay-ZUL-fih-deen) 1 2 dk | |  |  |
| c. Plaquenil (PLA-kwen-ill) or Hydroxychloroquine (hi-drox-ee-KLOR-oh-kwin) 1 2 dk | |  |  |
| d. Azathioprine (AY-zah-THIGH-oh-prin), Imuran (IM-your-an), or Azasan (AY-zah-sahn) 1 2 dk | |  |  |
| e. Arava (uh-RAVE-uh) or Leflunomide (leh-FLOON-oh-mide) 1 2 dk | |  |  |
| f. Xeljanz (ZEL-janz) or Tofacitinib (TOE-fah-SIT-in-ib) 1 2 dk | |  |  |
|  | |  |  |
| (**if =yes, add:** Other than the steroid injections you mentioned earlier) in the past 10 years have you ever been treated with any injectable medications for arthritis or autoimmune disease? 1 2 dk | |  |  |
|  | **(if = yes, ask:)** | | |
|  | Has this included . . . **(read in order until first “yes”, then skip to next)**? | |  |
|  | **yes no dk/ref** | |  |
|  | i. Enbrel (EN-brel) or etanercept (ee-TAN-er-cept) 1 2 dk | |  |
|  | ii. Humira (hugh-MEER-uh) or adalimumab (ah-duh-LIM-you-mab) 1 2 dk | |  |
|  | iii. Remicade (REM-ih-kaide) or Infliximab (in-FLIX-ih-mab) 1 2 dk | |  |
|  | iv. Simponi (sim-POHN-ee) or Golimumab (go-LIM-you-mab) 1 2 dk | |  |
|  | v. Cimzia (SIM-zee-ah) or Certulizumab (sert-uh-LIZ-oo-mab) 1 2 dk | |  |
|  | vi. Actemra (ack-TEM-rah) or Tocilizumab (toe-see-LIZ-oo-mab) 1 2 dk | |  |
|  | vii. Orencia (oh-REN-see-yah) or Abatacept (ab-AT-ah-sept) 1 2 dk | |  |
|  | viii. Rituxan (rih-TUX-an) or Rituximab (rih-TUX-ih-mab) 1 2 dk | |  |
